# A Novel Role for VICKZ Proteins in Maintaining Epithelial Integrity during Embryogenesis

**DOI:** 10.1371/journal.pone.0136408

**Published:** 2015-08-28

**Authors:** Michal Shoshkes Carmel, Nitza Kahane, Froma Oberman, Rachel Miloslavski, Dalit Sela-Donenfeld, Chaya Kalcheim, Joel K. Yisraeli

**Affiliations:** 1 Department of Developmental Biology and Cancer Research, The Faculty of Medicine, The Hebrew University of Jerusalem, Jerusalem, Israel; 2 Department of Medical Neurobiology, IMRIC, The Faculty of Medicine, The Hebrew University of Jerusalem, Jerusalem, Israel; 3 Koret School of Veterinary Medicine, The Hebrew University of Jerusalem, The Robert H. Smith Faculty of Agriculture, Food and Environment, 76100, Rehovot, Israel; University of Colorado, Boulder, UNITED STATES

## Abstract

**Background:**

VICKZ (IGF2BP1,2,3/ZBP1/Vg1RBP/IMP1,2,3) proteins bind RNA and help regulate many RNA-mediated processes. In the midbrain region of early chick embryos, VICKZ is expressed in the neural folds and along the basal surface of the neural epithelium, but, upon neural tube closure, is down-regulated in prospective cranial neural crest (CNC) cells, concomitant with their emigration and epithelial-to-mesenchymal transition (EMT). Electroporation of constructs that modulate cVICKZ expression demonstrates that this down-regulation is both necessary and sufficient for CNC EMT. These results suggest that VICKZ down-regulation in CNC cell-autonomously promotes EMT and migration. Reduction of VICKZ throughout the embryo, however, inhibits CNC migration non-cell-autonomously, as judged by transplantation experiments in Xenopus embryos.

**Results and Conclusions:**

Given the positive role reported for VICKZ proteins in promoting cell migration of chick embryo fibroblasts and many types of cancer cells, we have begun to look for specific mRNAs that could mediate context-specific differences. We report here that the laminin receptor, integrin alpha 6, is down-regulated in the dorsal neural tube when CNC cells emigrate, this process is mediated by cVICKZ, and integrin alpha 6 mRNA is found in VICKZ ribonucleoprotein complexes. Significantly, prolonged inhibition of cVICKZ in either the neural tube or the nascent dermomyotome sheet, which also dynamically expresses cVICKZ, induces disruption of these epithelia. These data point to a previously unreported role for VICKZ in maintaining epithelial integrity.

## Introduction

The cranial neural crest (CNC) forms at the border between the neural and non-neural ectoderm in the midbrain and hindbrain regions [1]. During neurulation in chick embryos, as the neural folds fuse dorsally, CNC cells delaminate and migrate to generate several cephalic structures that comprise mesectodermal and neural derivatives [2] (but see also [3]). In contrast, in Xenopus, CNC is never included in the neural tube (NT), but instead migrates from the border of the neural plate before NT closure (reviewed in [4]). Contrary to trunk levels of the axis where a complete epithelial-to-mesenchymal transition (EMT) of NC progenitors is required for the cells to engage in migration, CNC progenitors leave *en masse* and adopt first a collective mode of migration as a cohesive cell group that likely completes an EMT at the leading edge of the migratory population [2,4]. Initial directed CNC emigration involves planar cell polarity, which regulates polarized cell protrusions [5,6]. Subsequent interactions between CNC and placode cells have been shown to coordinate directed migration and morphogenesis [7]. Single cells at the leading edge of the CNC stream have been shown to adopt a unique molecular signature that has been proposed to help facilitate directed cell migration as well [8,9]. Although changes in cell adhesion molecules, transcription factors, and signaling pathways were shown to affect the onset of CNC migration and subsequent cell dispersion (cited in [2]), these highly dynamic and multistage processes are still not well understood.

The VICKZ proteins are a family of RNA binding proteins that mediate intracellular RNA localization, stability, translation, and splicing, in different cellular contexts [10,11]. The proteins are expressed during embryonic development in a wide range of cell types, generally undergo down-regulation after birth, and have been implicated in cell migration, cell proliferation, axonal guidance, and regeneration. Many cancers and neoplastic cell types up-regulate VICKZ proteins upon transformation, and expression of these proteins has been correlated with poor prognosis, poor overall survival, and/or metastasis in a large number of different types of tumors (e.g., [12–17]). A notable exception to this rule are metastatic mammary carcinomas, in which both *in vitro* and *in vivo* evidence suggest that VICKZ proteins are down-regulated in metastases and metastasizing cells [18,19]. In light of the large number of potential RNA targets identified for VICKZ proteins, it seems likely that their biological functions are diverse.

Previous work demonstrated that Vg1RBP (xVICKZ3) is expressed in the developing neural plate epithelium and the inner, sensory layer of the ectoderm in stage 17 Xenopus embryos, and throughout the closed NT and in the branchial arches in stage 21 embryos [20]. When xVICKZ3 expression was reduced throughout the embryo by injection of antisense morpholino oligonucleotides into both blastomeres of a 2-cell embryo, both cranial and trunk neural crest migration were inhibited [21]. These results indicated that VICKZ proteins play an important role in neural crest migration, yet the paradigm used, which affected multiple tissues, could not distinguish between cell autonomous vs. non-autonomous functions.

In the present study, we further investigated the connection between VICKZ proteins and CNC emigration in both chick and Xenopus embryos. We find that VICKZ is normally down-regulated during cell emigration and that loss or gain of VICKZ activity in the neural epithelium promote or inhibit the exit of CNC cells from the NT, respectively. In addition, VICKZ protein appears to be an important factor in maintaining epithelial integrity in the embryo, as prolonged expression of an inhibitor of VICKZ activity disrupts the epithelial morphology of both the NT and dermomyotome and modulates expression of integrin α6, a laminin receptor. These results suggest that VICKZ proteins play a role in maintaining the epithelial state of various embryonic tissues. Together, this illustrates that VICKZ proteins can have complex and diverse effects on cell behavior, depending on the context in which they are expressed.

## Materials and Methods

### Ethics Statement

Fertilized, White Leghorn eggs were purchased from Gil-Guy (Moshav Orot, Israel). No avian breeding is carried out in our animal facilities. No formal approval is required by the Faculty of Medicine of the Hebrew University to perform experiments on early avian embryos (2–5 days of incubation). All amphibian work was conducted according to relevant national and international guidelines. Animal protocols were approved by the Hebrew University Ethics Committee.

### Manipulation and Injection of Xenopus Embryos

Xenopus eggs were stripped, fertilized and injected as previously described [22]. Embryos were maintained in 0.1X Modified Barth’s Solution-HEPES (MBSH) and, at the two-cell stage or 32-cell stage for the blastomere specific injections, they were transferred to 1X MBSH for injection. Following 30min incubation at room temp, they were transferred to 17°C, where they were maintained in 0.1X MBSH until the desired stage was reached. Morpholino Oligonucleotides either directed against a sequence in the 5' UTR of Vg1RBP (5'AAAGAAGACGAGCCCGAAAAACCCG3') or encoding a control sequence (5'CCTCTTACCTCAGTTACAATTTATA3'), were purchased from Gene Tools LLC, resuspended in sterile, filtered water, and10ng/blastomere were injected. For the blastomere specific injections 5ng of fluorescinated-dextran was co-injected with 10ng of the morpholinos.

### Xenopus CNC Transplantation Assay

CNC transplantations were performed as described [23]. GFP mRNA was synthesized by *in vitro* transcription of SP6 using the Cap Scribe RNA kit (Roche). Injection of AMO was coupled with Texas-Red Alexa dextran (Molecular Probes).

### *in-Ovo* Electroporation of Chick Embryos

Fertile chick (*Gallus gallus*) eggs from Gil-Guy (Moshav Orot, Israel) were used in this study. The chicken full-length cVICKZ1 (ZBP-1) and a point mutation construct Y396F were subcloned into the pCAGGS-IRES-GFP backbone to generate pCAGGS-cVICKZ1-IRES-GFP and pCAGGS-Y396F-IRES-GFP, respectively. 3μg of pCAGGS-IRES-GFP control vector, cVICKZ1-IRES-GFP and Y396F-IRES-GFP DNA were microinjected into the midbrain lumen of 2–4 somite stage (ss) embryos and to the center of flank-level epithelial somites at E2.0 (HH15). To enable dorsal/lateral labeling of the hemi-tube, electroporation was performed with bent L-shaped gold electrodes (1 mm diameter) in a parallel holder (BTX, Harvard Apparatus) positioned above the embryo on the left and right sides of the midbrain neural folds. Three 50-ms pulses of 16V and pulse intervals of 1sec were applied using a BTX electroporator (ECM830). For the dermomyotome, electroporations were performed on the dorsal part of the epithelial somite, as previously described [24]. A square wave electroporator (BTX, San Diego, CA, USA) was used to deliver a single pulse of 20V for 10ms.

### Electroporation of Antisense Morpholino Oligonucleotides

A fluorescein-tagged antisense morpholino oligonucleotide (AMO) directed against all three of the cVICKZ paralogs was designed by targeting the first 25 nucleotides of the cVICKZ1 open reading frame that has only 2 mismatch with the other two mRNA paralogs (Gene Tools, LLC). The antisense sequence used was: 5'GGTTCCCGATGTACAGCTTGTTCAT3'. The working concentration of cVICKZ-AMO and of a standard control morpholino oligonucleotide (CMO) was 1mM. Morpholinos were injected into the midbrain lumen of 2-4ss embryos as described for the DNA construct injections. To allow prolonged incubation with the AMO, post electroporation embryos were kept at room temperature overnight, incubated for 2–3 hours at 42°C, and then fixed at the 8 somite-stage. To test the efficacy of the AMO, a chick mesenchymal stem cell line (a gift from Professor Joseph Yanai, Department of Medical Neurosciences, Hebrew University) was incubated for 48 hours with either 2.5 or 10μM AMO and Endoporter (Gene Tools LLC), according to manufacturer’s instructions, to enhance uptake of the AMO into cells. Protein cell lysates were analyzed by western blotting using anti-panVICKZ and anti-actin antibodies and quantified using Image J software.

### Embryo Processing and Immunofluorescence

Embryos were fixed with 4% formaldehyde, wax-embedded and sectioned at 5μm or observed as whole mount preparations. Antibodies used were: anti-Desmin and anti-GFP (Molecular Probes), N-cadherin and ZO-1 (Zymed), anti-integrin α6 and anti-snail2 (P2C62C4 and 62.1E6, Developmental Studies Hybridoma Bank), affinity purified anti-panVICKZ [16,17] and HNK-1 (BD Pharmingen San-Diego, CA). Polyclonal antibody against ZO-1 and monoclonal antibodies against N-cadherin and integrin α6 were applied following antigen retrieval by boiling the slides in 0.1M Tris buffer (pH 9.5) for 10 minutes.

### RT-PCR

Total RNA was isolated from different stages chick embryos using TRIzol RNA purification kit (Ambion) according to manufacturer’s instructions. Reverse transcriptase reaction was performed using Moloney Murine Leukemia Virus Reverse Transcriptase (M-MLV RT; Invitrogen) using random primers according to manufacturer’s instructions. Expression of cVICKZ 1, 2, and 3 isoforms was measured by RT-PCR using the primers:

cVICKZ1Forward (F): 5'GGAGGAAATGAGAGCAGCAC3'

cVICKZ1Reverse (R): 5'TTCTTAGCCCCATCCCTCTT3'

cVICKZ2 F: 5'CAGCCTCCACCTGACTTGAT3'

cVICKZ2 R: 5'ATGTATGACCAGCACGGACA3'

cVICKZ3 F: 5'CCTTTGTGCCTTTGACCATCACTAC3'

cVICKZ3 R: 5'AATTAACCCTCACTAAAGGGCTGTTA3'

As positive controls, we used a ZBP1plasmid kindly provided by Rob Singer for cVICKZ1, and the Rinken15e15 plasmid (accession number AJ719686) kindly provided by H. Arakawa for cVICKZ3.

### RNA Immunoprecipitation and Quantitative RT-PCR

Lysates were prepared from 3d and 4d embryos, and RNA-protein complexes (RNPs) were IP’d with 5 ug of either pre-immune serum or anti pan-VICKZ antibody, using Protein A Dynabeads (Life Technologies), according to the RIP method described in Jayaseelan et al. [25]. Total and IP’d RNAs were isolated using the EZ-RNA Isolation Kit (Biological Industries) and reverse-transcribed with random primers using the ImPromII Reverse Transcription System (Promega) in 20ul reactions. cDNAs were analyzed by quantitative PCR reaction using the iQ SYBR Green Supermix and the iCycler system (Bio-Rad). Primers used for the amplification were as follows: ITGA6: forward 5’- TTGGAGCCCCGCAATACTTT-3' and reverse, 5’- AGCGGTCCCATTTAAGCGAA -3’; 18S: forward 5’- TGTGCCGCTAGAGGTGAAATT -3’ and reverse, 5’- TGGCAAATGCTTTCGCTTT -3’. Quantitative RT-PCR reactions were done in triplicate.

### Data Analysis and Statistics

The proportion of GFP-positive cells exiting and migrating from the neural tube was calculated by dividing the GFP-positive cells outside of the tube by the total number of GFP-positive cells (calculated as the number of GFP-positive cells within a circumscribed 45° arc from the dorsal midline on the transfected side in the dorsal neural tube plus those outside the tube). CNC delamination was analyzed in 5 embryos per treatment, with 4–6 sections per embryo analyzed. Results represent the average proportion of cells exiting the tube per embryo (±SEM). Significance was determined using an unpaired t-test.

## Results

### Down-Regulation of cVICKZ in the Dorsal Neural Tube Is Associated with the Onset of CNC Delamination

**Fig 1:**
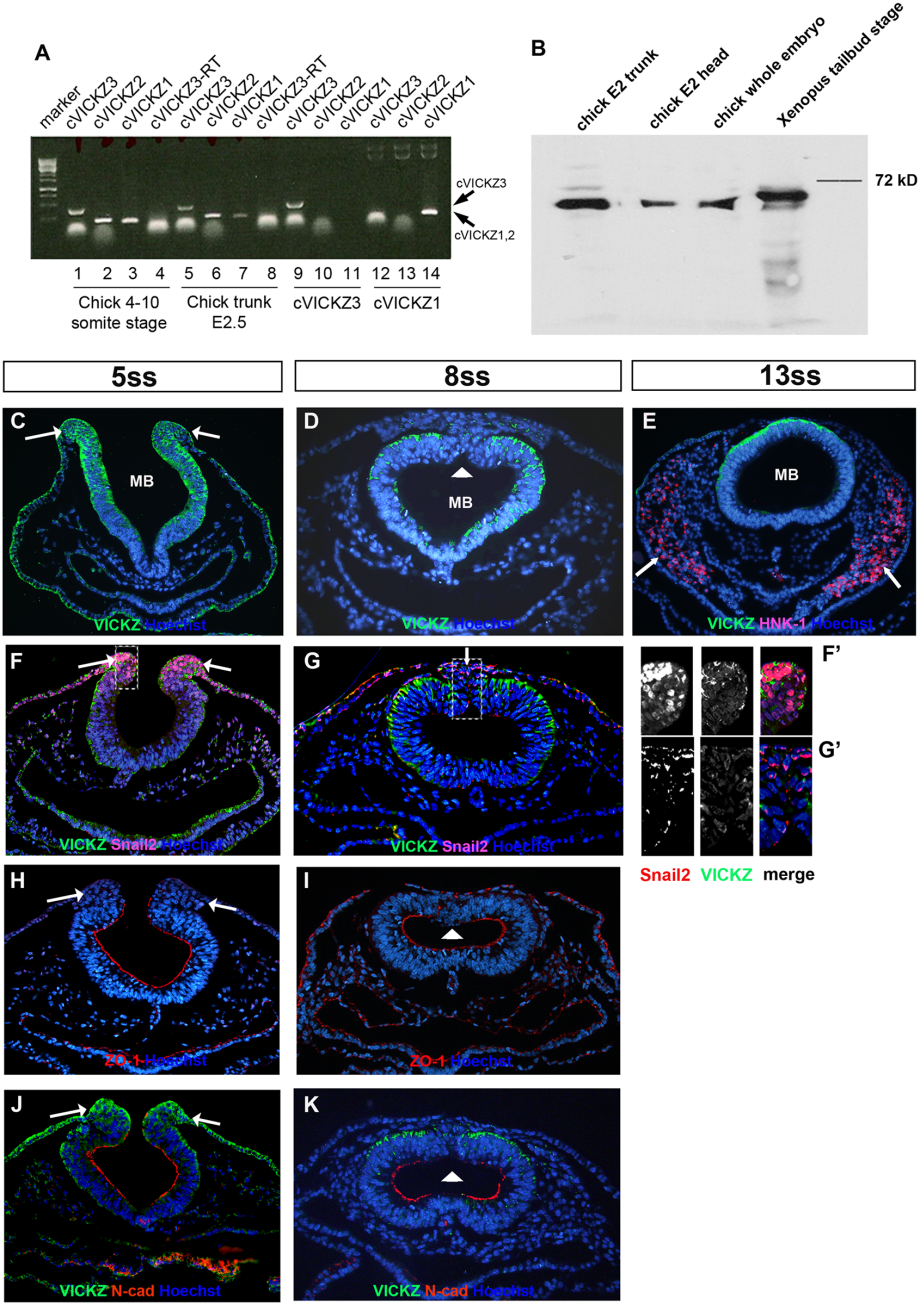
VICKZ expression during CNC delamination. (A) The three VICKZ paralogs were detected by RT-PCR in isolates from chick embryos at the 4–10 somite-stage (lanes 1–4) and at E2.5 (trunk region, lanes 5–9), using primer sets for cVICKZ3 (lanes 1,5), cVICKZ2 (lanes 2,6) and cVICKZ1 (lanes 3,7). All RT-PCR products were the appropriate size on ethidium bromide stained agarose-gel (cVICKZ3 = 426nt, cVICKZ2 = 206nt, cVICKZ1 = 172nt). Marker-1kb ladder. Negative controls: cVICKZ3-RT, PCR reaction using cVICKZ3 primers but without Reverse Transcriptase (lanes 4,8). Positive controls: cVICKZ3 (clone 5e15, Pubmed accession number AJ719686; lanes 9–11) and ZBP-1 for cVICKZ1 (lanes 12–14) using primer sets for cVICKZ3 (lanes 9,12), cVICKZ2 (lanes 10,13) and cVICKZ1 (lanes 11,14). (B) Proteins were extracted from tailbud stage *Xenopus* embryos, or from chick E2 stage whole embryos, head, or trunk. One embryo equivalent was loaded on each lane, and VICKZ expression was examined following electrophoresis by western blot analysis using an anti-pan VICKZ antibody. (C-K) Cross-sections at the midbrain (MB) level in chick embryos at 5ss (C,F,H,J), 8ss (D,G,I,K), and 13ss (E) were stained using antibodies directed against VICKZ (C-G, J-K), HNK-1 (E), Snail2 (F-G), ZO-1 (H-I), and N-cadherin (J-K). (F’) and (G’) are higher magnifications of the individual channels (red, green, and merge) of the boxed regions in (F) and (G). At early stages of CNC specification, presumptive CNC cells are located in the neural folds in the dorsolateral region (C, F, F’, H, J, arrows), where they express both VICKZ and Snail2 (F). ZO-1 and N-cadherin are expressed along the apical surface of the neuroepithelium at this stage, but are mostly excluded from the neural folds (H,J). Down-regulation of VICKZ occurs in 8ss embryos in the dorsal-most, delaminating CNC cells that remain Snail2-positive (G, arrow, G’) and are negative for ZO-1 and N-cadherin (I,K arrowhead). HNK-1-positive migrating CNC do not express VICKZ (E, arrows). Abbreviations: MB, midbrain. All sections were also stained with Hoechst to show nuclei (blue).

Previous work in Xenopus indicated that xVICKZ is required for neural crest migration [21]. To better understand the role VICKZ plays during this process, we turned to the chick embryo as a model system in which the effects of reducing VICKZ activity within specific cells can be analyzed in the context of an otherwise wild type embryo. RT-PCR indicated that all three VICKZ (cVICKZ) paralogs are expressed in chick embryos (Fig 1A). An affinity purified polyclonal antibody against Xenopus xVICKZ3 that recognizes all three paralogs (anti-panVICKZ) detected only a single band of the expected molecular weight on a Western blot (Fig 1B). The emigration of CNC cells from the NT, described as an EMT, is known to involve the orchestrated expression of a set of genes. In order to define how cVICKZ relates to the process of EMT, we compared its expression to known EMT markers such as Snail2, ZO-1, and N-cadherin. During early stages, before any morphological evidence of EMT is observed, Snail2, ZO-1, and N-cadherin all define prospective CNC in the neural folds, with Snail2 expressed in, and ZO-1 and N-cadherin excluded from, these cells (Fig 1F, 1H and 1J, arrows). cVICKZ is expressed throughout most of the neural epithelium and particularly in the neural folds at these stages (Fig 1C, 1F, 1F’ and 1J, arrows). Its expression is tightly, although not exclusively, associated with the basal surface of the cells, while ZO-1 and N-cadherin are localized to the apical surface (Fig 1H and 1J). Concomitant with CNC delamination, cVICKZ expression is strikingly down-regulated specifically in the dorsal midline region of the midbrain epithelium and in the emigrating Snail2 positive, ZO-1 and N-cadherin negative, cells (Fig 1D, 1I and 1K, arrowhead; G, arrow G’). These CNC cells remain cVICKZ negative throughout migration but become HNK-1 positive as they migrate (Fig 1E, arrows). Thus, EMT and CNC emigration appear to be tightly correlated with the cVICKZ down-regulation that occurs at the 8ss.

### Inhibition of cVICKZ Activity Enhances CNC Delamination and Causes Epithelial Dissociation

**Fig 2:**
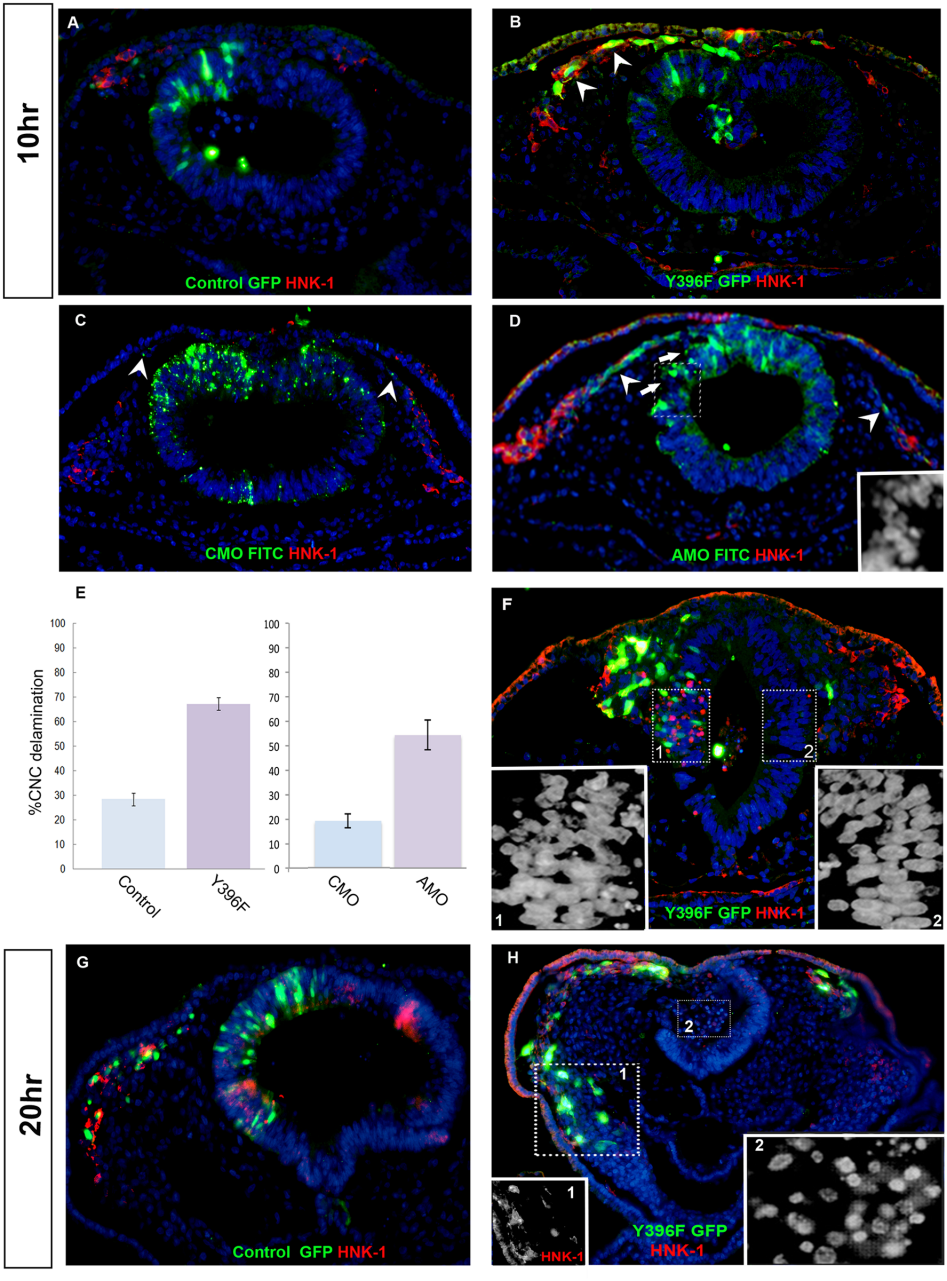
Down-regulation of VICKZ enhances CNC delamination and causes NT dissociation. 2-4ss embryos were electroporated with either control GFP (A,G), Y396F-GFP (B,F,H), FITC labeled control morpholino (CMO) (C) or FITC labeled cVICKZ-antisense morpholino (AMO) (D) and fixed and sectioned either 10 hours (A,B,C,D,F) or 20 hours (G,H) post-electroporation. All sections were stained for HNK-1 expression (red) and GFP (green). (E) For each treatment, the percentage of the total number of GFP-positive cells that delaminated post-electroporation was calculated for each embryo (see Materials and Methods) and graphed as the mean ± SEM (N = 5). Y396F-treated embryos showed a 2.4-fold increase in delamination as compared to the controls; AMO-treated embryos showed a 2.8-fold increase in delamination compared to CMO-treated embryos. Insets in D, F and H (inset 2) show enlarged images of the nuclei in the corresponding rectangles indicated by the dotted lines. Inset 1 in H shows the red channel (HNK-1) outlined by the rectangle. Arrowheads indicate emigrating, electroporated CNC (B,C,D). Arrows in (D) indicate disorganization of the electroporated neuroepithelium.

To test whether down-regulation of cVICKZ could be causally related to CNC EMT, we made use of chick *in-ovo* electroporation to introduce various constructs into a limited number of cells in the developing neural epithelium. Src phosphorylation of cVICKZ1 at Tyr396 has been shown to be critical for relieving cVICKZ1-mediated translational repression of β-actin mRNA by inducing release of the target mRNA from the protein [26,27]. A point mutation that renders the molecule non-phosphorylatable at that site (Y396F) acts as a dominant negative construct, inhibiting translation by maintaining bound RNA, in both non-neuronal and neuronal cells [26–28]. Y396F-GFP (Y396F) or control-GFP were transfected into dorsal hemi-neural folds of embryos aged 2–4 somite pairs at the level of the midbrain. At 10 hours post-electroporation, only a few HNK1-positive CNC cells are observed outside of the neural tube in control embryos (Fig 2A). Electroporation of the Y396F-GFP construct, however, strongly enhanced the number of emigrating HNK1-positive CNC cells, as compared to the control GFP construct (Fig 2B). In addition, most of the HNK1-positive cells on the Y396F-GFP transfected side are GFP-positive, suggesting that the effect of the construct is cell-autonomous. In order to quantify the effect, the fraction of migrating GFP-positive cells was calculated (see Materials and Methods). Y396F enhanced CNC emigration 2.4-fold at 10 hours post-electroporation, as compared to the GFP control (Fig 2E).

We further validated the role of cVICKZ through the use of an antisense morpholino nucleotide (AMO) to knock down cVICKZ. Because all three cVICKZ paralogs are expressed during chick embryogenesis (Fig 1A), we designed an AMO against the first 25 nucleotides of the cVICKZ1 open reading frame that was predicted to also bind the other two paralog mRNAs (only two mismatches; see Materials and Methods). To confirm specificity, chick mesenchymal stem cells were incubated with this AMO, and VICKZ protein was assayed by western blot. VICKZ expression was reduced by approximately 40% by the AMO, when normalized to actin (S1 Fig). Morpholino oligonucleotides, labeled with FITC, were injected into the midbrain lumen of 2-4ss embryos, electroporated and processed as described in Materials and Methods. As seen in S2 Fig, cells that received the AMO show reduced expression of VICKZ, indicating its efficacy in vivo. Consistent with the results observed with the Y396F construct, cVICKZ-AMO similarly enhances emigration of GFP-labeled progenitors when compared to control morpholino-treated embryos (Fig 2C and 2D). These results were quantified as with the Y396F construct, and the AMO knockdown of cVICKZ shows a 2.8-fold increase in the percent of CNC delamination over embryos electroporated with CMO (Fig 2E).

In the AMO-injected embryos, we noticed not only that the majority of the CNC cells migrating outside of the tube had received the AMO, but also that the AMO-containing cells retained in the lateral domain of the neural epithelium appeared to be poorly organized (Fig 2D, arrows). When the embryos transfected with the Y396F construct were more carefully examined, some of these embryos also showed a similar disruption of the pseudostratified epithelium on the electroporated side of the lateral neural tube. As seen in Fig 2F, nuclei on the transfected side of the tube, at 10 hours post-electroporation, that were located outside of the dorsal region (inset 1), appeared to be more rounded and disorganized than those in the comparable region on the uninjected side (inset 2). At 20 hours post-electroporation, there was a complete dissociation of the labeled epithelium with many pyknotic cells present in the lumen of the Y396F-transfected neural tube (Fig 2H). In contrast, GFP-labeled cells were retained in the control-injected tubes and maintained their normal epithelial appearance (Fig 2G). Significantly, HNK1-positive Y369F-GFP labeled CNC cells continued on their ventral migration (Fig 2H, inset 1), suggesting that the Y396F construct did not affect the migration of neural crest cells, but rather the integrity of the neural epithelium.

### Overexpression of cVICKZ Inhibits CNC Delamination

**Fig 3:**
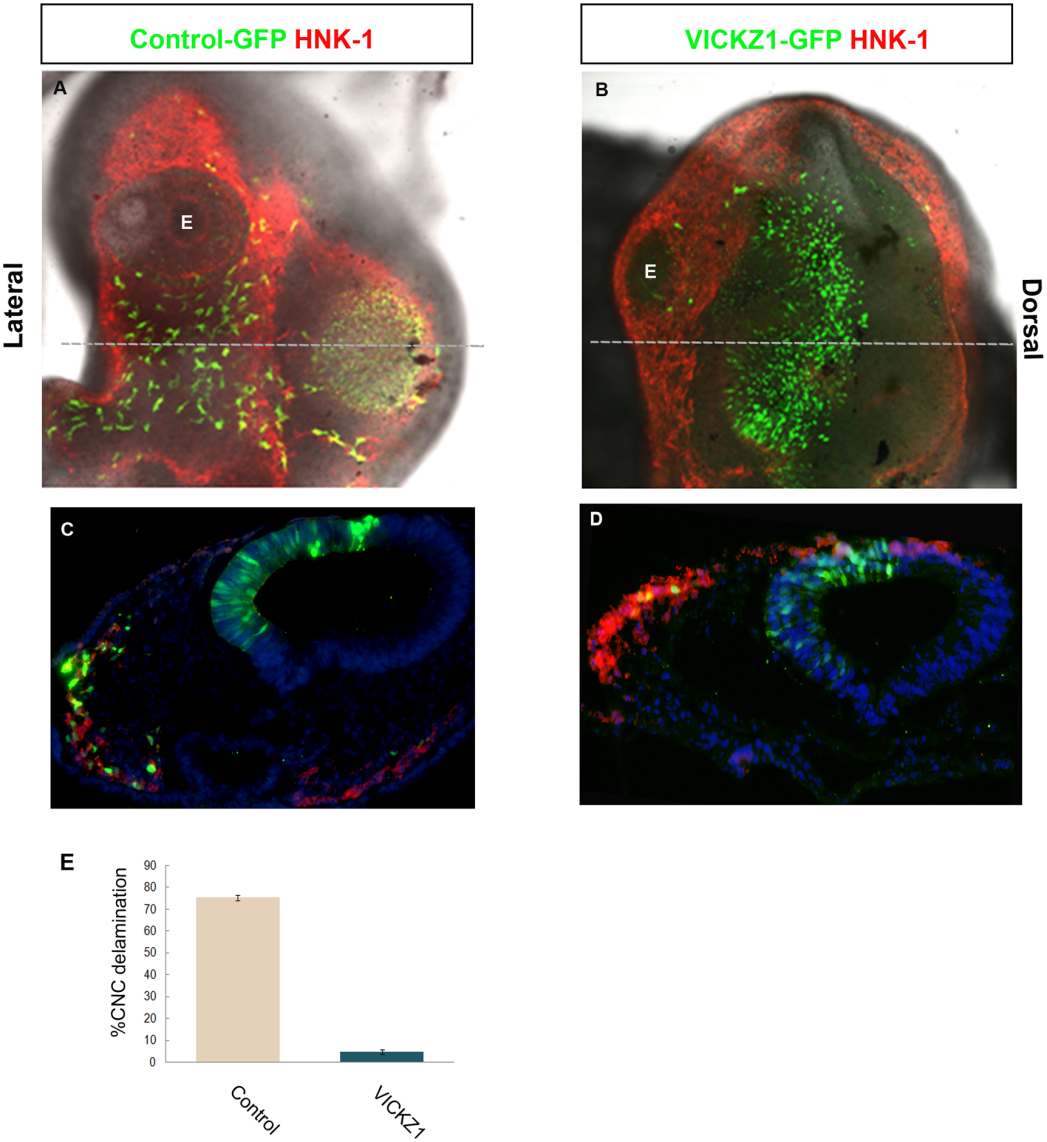
Overexpression of cVICKZ1 inhibits CNC emigration. 2-4ss embryos were electroporated with either control GFP (A,C) or cVICKZ1-GFP (B,D), and fixed 20 hours post-electroporation. Embryos were stained for HNK-1 expression (red) and GFP (green) and photographed either as whole mount preparations (A,B) or after sectioning (C,D). The grey dotted line in (A) and (B) indicate the plane of section shown in (C) and (D), respectively. Almost no CNC delamination occurs in the cVICKZ1-GFP embryos, although GFP-negative, HNK-1-positive cells emigrate normally. (E) The percentage of the total number of GFP-positive cells that delaminated by 20 hours post-electroporation was calculated for each embryo and was graphed as the mean ± SEM (N = 5). Abbreviations: E, eye.

Our results indicate that cVICKZ plays an important role in maintaining epithelial integrity in the neural tube and that down-regulating cVICKZ in the dorsal neural tube enhances CNC delamination. These findings suggest that it is sufficient to reduce cVICKZ activity in order to allow CNC progenitors to emigrate. If this down-regulation is also necessary for CNC EMT, then overexpression of cVICKZ in the dorsal neural tube should delay or prevent CNC delamination. We therefore electroporated a plasmid expressing full-length cVICKZ1 into the hemi-tube at the midbrain level as described earlier and analyzed the embryos 20 hours post-electroporation, a time when a substantial proportion of CNC in control embryos has already emigrated (see Fig 2G). In whole mount embryos electroporated with control GFP, CNC cells are observed to delaminate and migrate along their normal pathways (Fig 3A; lateral view). In contrast, embryos electroporated with full length cVICKZ1 maintain the GFP-positive CNC within the neural tube, and they show very little if any delamination (Fig 3B; dorsal view). Quantitative analysis of sectioned embryos confirmed this effect, as full-length cVICKZ1 in prospective CNC cells reduced their overall emigration by over 70%, with these cells almost completely failing to delaminate as compared with cells that received control-GFP (Fig 3C, 3D and 3E). Therefore, cVICKZ expression inhibits CNC delamination, and its normal down-regulation is necessary for the onset of CNC emigration.

### Down-Regulating cVICKZ Causes Dermomyotome Dissociation

**Fig 4:**
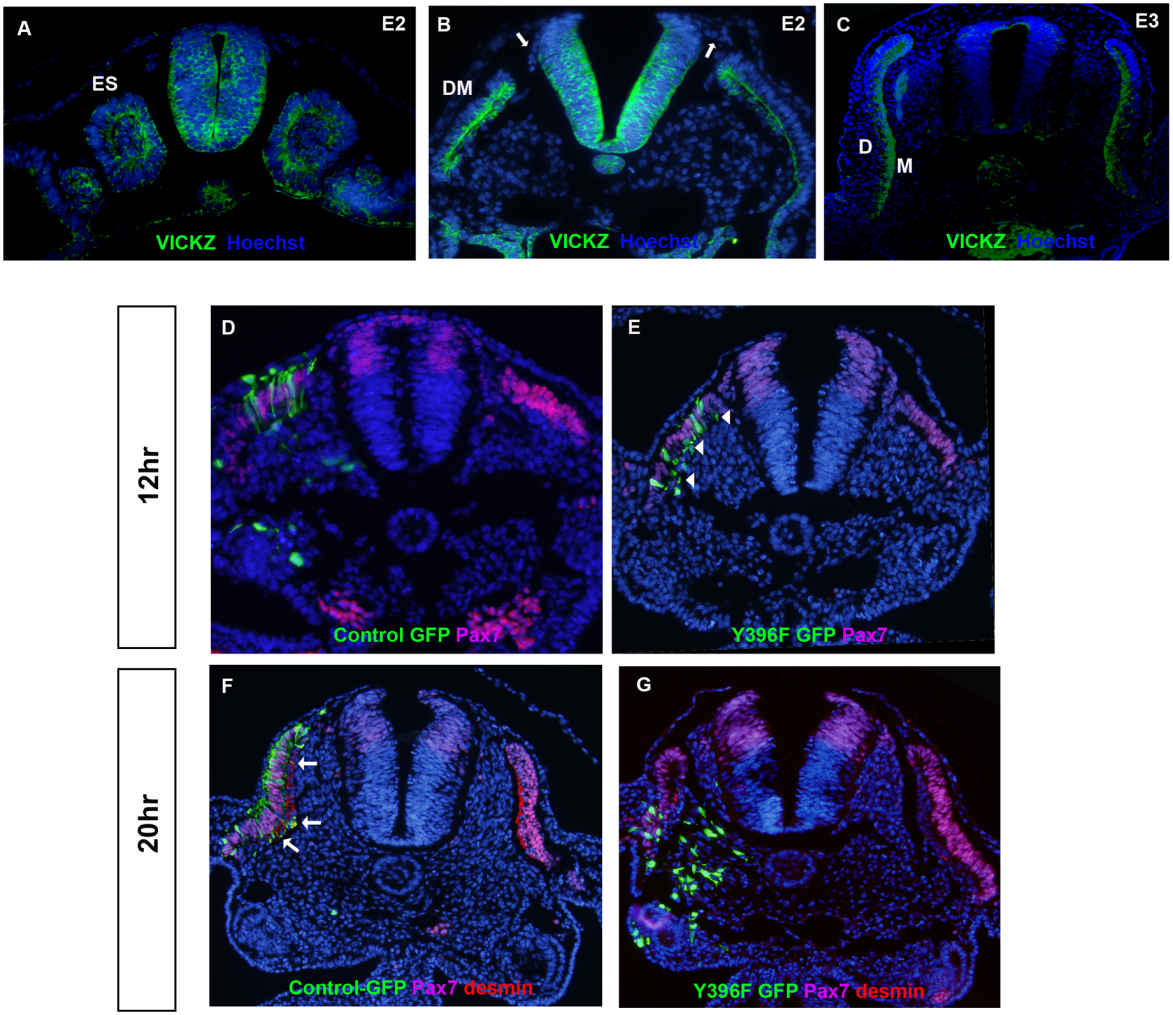
Down-regulation of VICKZ causes DM dissociation. (A-C) VICKZ is expressed in newly-formed somites in E2 embryos (A) particularly in the apical adherens junctions, and later in the early dermomyotome (B). In E3 embryos, VICKZ is down-regulated in the emerging dermis but maintained in the developing myofiber (C). (VICKZ is also down-regulated in the emigrating trunk NC cells (arrows in B)). (D-G) Embryos were electroporated with either control GFP (D, F) or Y396F-GFP (E, G) into the dorsal medial lip (DML) of early dissociating somites. Embryos were fixed either 12 (D,E) or 20 (F,G) hours post-electroporation and stained for the DM marker, Pax 7 (purple) and/or the myotome marker, Desmin (red). After 12 hours, control GFP-labeled DML cells retain their epithelial structure and are Pax7-positive (D) whereas many DML cells that received Y396F-GFP have begun rounding up and losing their epithelial pseudostratified morphology (E, arrowheads). By 20 hours post-electroporation, in the control-GFP embryos, GFP-positive, Desmin-positive myofibers derived from the DML occupy the medial-most aspect of the myotome (F, arrows). In Y396F-GFP treated embryos, however, the DM was completely dissociated, with GFP-positive, Pax7-negative cells having lost their epithelial morphology. None of the GFP-positive cells generated myofibers. Abbreviations: ES, epithelial somite; DM, dermomyotome; D, dermis; M, myotome.

To better understand whether the observed effects of cVICKZ on epithelial integrity are limited to the neural tube or more wide-spread, we looked for other epithelia in which cVICKZ is expressed and could be perturbed by electroporation. The dermomyotome (DM), derived from the dorsal half of the epithelial somite, is a transient epithelium lying in between the sclerotome and the surface ectoderm [29]. Following EMT, the central DM sheet mainly dissociates into two distinct populations of cells: mesenchymal cells that colonize the sub-ectodermal space to generate dermis, and the myotome that gives rise to vertebral muscles. During somite development, cVICKZ is expressed by cells in the epithelial somite, with predominant staining at the apical surfaces and weaker expression in the somitocoele (Fig 4A). The young DM retains strong apical cVICKZ expression (Fig 4B). However, upon DM dissociation, cVICKZ protein is down-regulated in the emerging mesenchymal dermis cells, while it remains stably expressed in myofibers (Fig 4C). Given the down-regulation of cVICKZ expression in the prospective cells undergoing EMT, we wondered whether cVICKZ might also play a role in maintaining the dermomyotome epithelium, as it does in the neural tube. To examine this possibility, the nascent dermomyotome sheet was electroporated with plasmids expressing either control-GFP or Y396F-GFP. Twelve hours post-electroporation, the majority of control GFP-expressing cells are still part of the dermomyotome epithelium and co-express the progenitor marker Pax7 (Fig 4D). Among the cells expressing Y396F-GFP, however, the beginning of de-epithelialization can be observed, with many of the GFP-positive cells no longer expressing Pax7 (Fig 4E arrowheads). By 20 hours, some control-GFP electroporated cells are still confined to the DM epithelium, while others have begun dissociating into dermis or generating labeled cells in myotome (Fig 4F arrows). In contrast, a dramatic dissociation of Y396F-transfected DM cells is observed (Fig 4G). Cells that have lost their epithelial structure appear to be spread over the adjacent mesoderm and are all negative for both Pax7 and desmin. These data suggest that, as in the case of the neural tube, cVICKZ plays a positive role in maintaining the DM epithelium.

### Integrin α6 Is Down-Regulated during CNC Delamination and Is Downstream of cVICKZ

**Fig 5:**
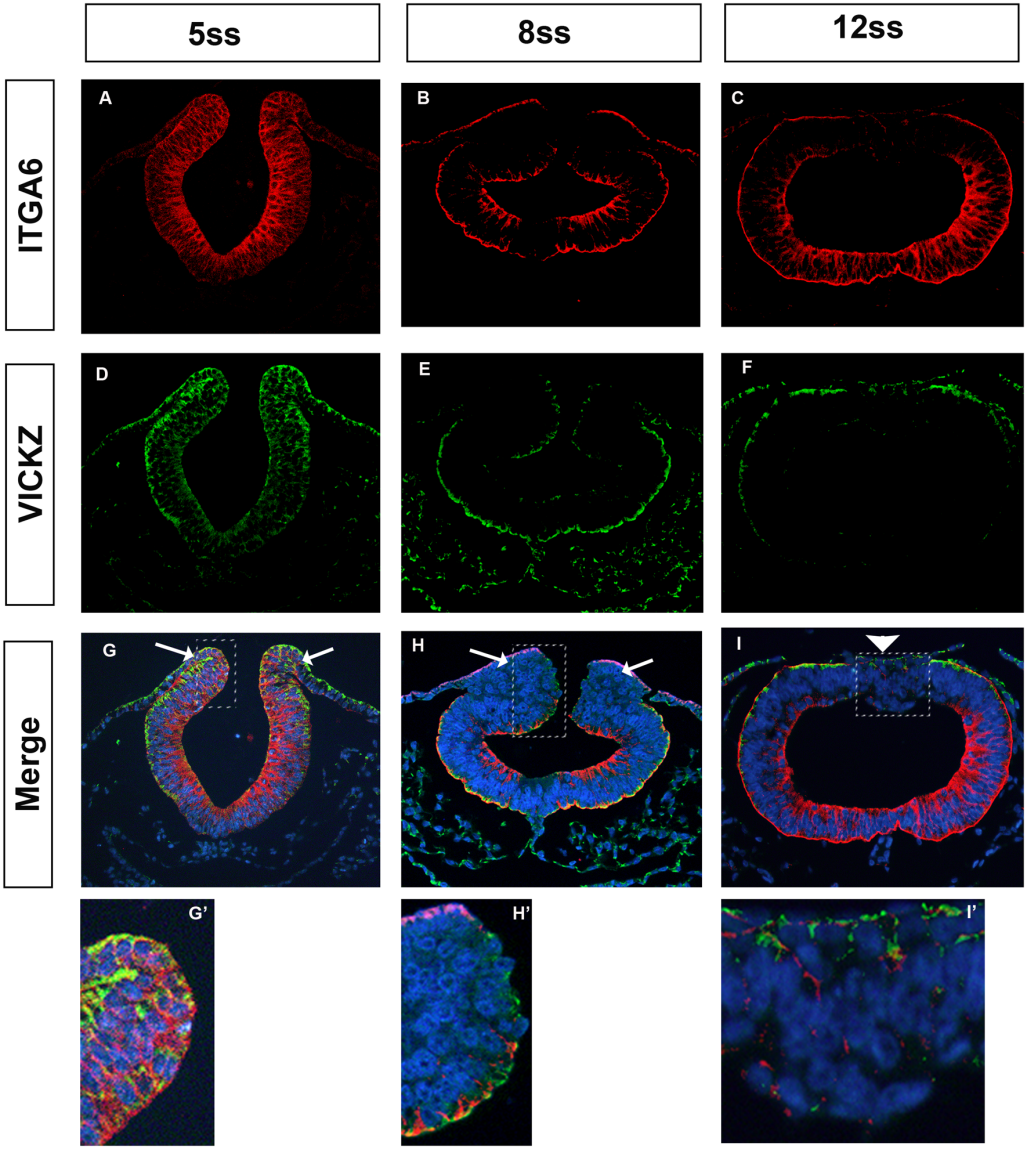
Integrin α6 and VICKZ expression during CNC delamination. Cross-sections at the midbrain level from chick embryos at 5ss (A,D,G), 8ss (B,E,H), and 12ss (C,F,I) were double stained with antibodies against Integrin α6 (A-C) and VICKZ (D-F). Merged images are shown in panels G-I. Higher magnification of the regions indicated by rectangles in G-I are shown in G’-I’. Integrin α6 is co-expressed with VICKZ in the neural folds in 5ss embryos (G, arrows, G’), undergoes down-regulation concomitantly with VICKZ as CNC delaminate (H, arrows, H’), and remains down-regulated, as does VICKZ, immediately following CNC emigration (I, arrowhead, I’).

**Fig 6:**
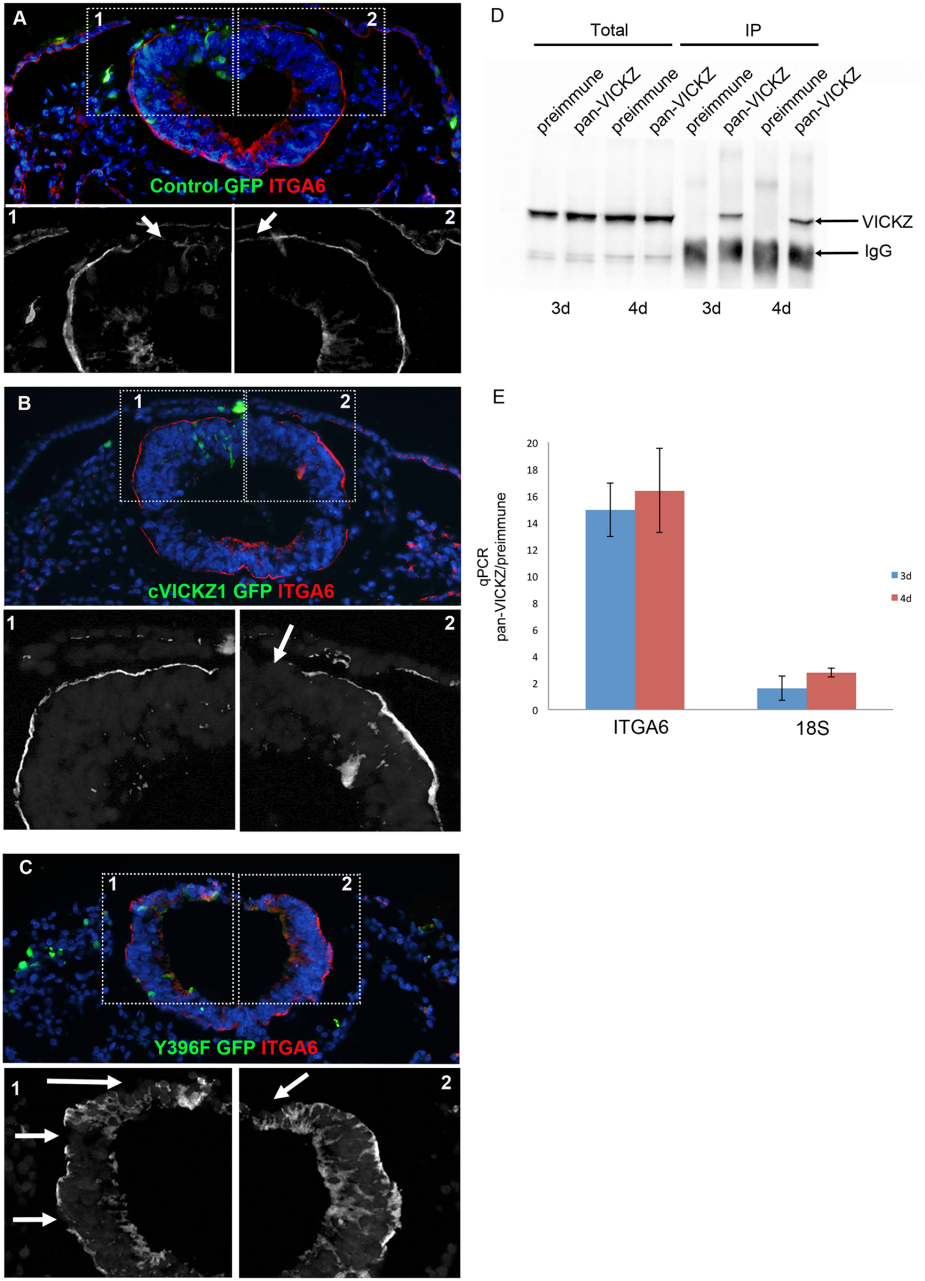
VICKZ1 mediates Integrin α6 expression and co-immunoprecipitates with Integrin α6 mRNA. 2-4ss embryos were electroporated with control-GFP (A), full length VICKZ1- GFP (B), or Y396F-GFP (C), and then fixed and stained for Integrin α6 (red) and GFP (green) 10 hours later. In control-GFP transfected embryos, Integrin α6 is down-regulated in the dorsal aspect of the tube in the region where CNC delaminate, on both the transfected and non-transfected sides (A, inset 1 and 2). Overexpression of VICKZ1 maintains Integrin α6 expression even in the most dorsal regions of the tube, but only on the transfected side (B, inset 1), and not on the non-transfected side (B, inset 2). Y396F expression causes a precocious emigration of CNC and down-regulation of Integrin α6 in more lateral regions of the tube, only on the transfected side (C, inset 1) and not on the non-transected side (C, inset 2). All insets show only the red channel (ITGA6). Arrows in insets indicate areas of downregulation of ITGA6. (D) RNP complexes were prepared from 3d and 4d old chick embryos and immunoprecipitated with either pre-immune serum or pan-VICKZ antibody. Equal volumes of total lysates (Total) and immunoprecipitates (IP) were subjected to western blot analysis using the pan-VICKZ antibody. VICKZ protein is pulled down exclusively by the pan-VICKZ antibody. (E) Quantitative RT-PCR analysis was performed on cDNAs prepared from both pre-immune and pan-VICKZ immunoprecipitations and tested for the presence of ITGA6 mRNA. Values of pan-VICKZ IP mRNA were normalized to the amounts of total mRNA, and compared to the pre-immune normalized values (pan-VICKZ/pre-immune). A 15–16 fold enrichment of ITGA6 mRNA is observed in the pan-VICKZ IP, as compared to the pre-immune serum. A control RNA, 18S, shows only 1–2 fold enrichment when analysed in the same way. The data show the mean±SEM.

Our results show that VICKZ expression is negatively correlated with EMT and plays a role in maintaining epithelial integrity. Its expression in epithelial structures is strikingly localized to either the basal or apical domains of cells. The localization of cVICKZ to the basal side of the cranial neural epithelium raised the possibility that it may be involved in mediating the expression of mRNAs encoding cell adhesion molecules, much as muscle-blind-like protein (MLP1) has been proposed to localize Integrin α3 mRNA [30]. Previously, Integrin α6 was shown to be expressed during neural tube closure and on premigratory neural crest cells at the midbrain level of stage 8 chick embryos, being predominantly expressed on the basal side of the neural epithelium [31]. We reasoned that its mRNA could serve as a potential target of VICKZ, and, if Integrin α6 were down-regulated during EMT, could play a role in CNC cell delamination. First, we compared Integrin α6 and VICKZ expression patterns during CNC development. During neural tube closure, Integrin α6 was abundantly expressed throughout the neuroepithelium, including premigratory CNC cells (Fig 5A, 5G, arrows, G’), as reported previously [31]. As the CNC cells emigrate, however, Integrin α6 is down-regulated at the dorsal-most, basolateral side of the neural tube and in delaminating CNC cells (Fig 5B, 5H arrows H’), and this down-regulation is maintained for several hours (Fig 5C, 5I and 5I’). This pattern of expression and down-regulation echoes that seen with VICKZ protein at the basal side of the neural epithelium and tube (Fig 5G–5I; compare G,H, arrows and I, arrowhead, and G’, H’, and I’). To test whether VICKZ actually modulates integrin α6 expression, we either overexpressed or down-regulated VICKZ in the dorsal neural tube and analyzed Integrin α6 expression. Ten hours post-electroporation, in embryos that received only the GFP control, Integrin α6 is down-regulated in the dorsal-most, basolateral region of the neural tube, just as is seen in untreated embryos undergoing CNC delamination (Fig 6A). Overexpression of cVICKZ1, however, results in an abnormal maintenance of Integrin α6 expression precisely on the transfected side of the neural tube (Fig 6B, inset 1), while the non-transfected, control side down-regulates the protein (Fig 6B, inset 2), as observed in both the control-GFP (Fig 6A) and untreated embryos (Fig 5B and 5C). Conversely, down-regulation of VICKZ activity by the Y396F construct broadens the down-regulation of basolateral Integrin α6 expression to more ventral regions in the neural tube (Fig 6C, inset 1, arrows), while not affecting Integrin α6 expression on the untreated side (Fig 6C, inset 2). Because Integrin α6 expression tightly correlates with VICKZ levels in the developing midbrain neural epithelium, and is mediated by it, these results suggest that VICKZ acts upstream of Integrin α6, either directly or indirectly, to regulate its expression.

To test more directly whether VICKZ interacts with ITGA6 mRNA, we performed co-immunoprecipitation from 3d and 4d old embryos using the pan-VICKZ antibody, and tested for the presence of ITGA6 mRNA using quantitative RT-PCR (Fig 6D and 6E). The pan-VICKZ antibody enriches ITGA6 mRNA concentration 15–16 fold when compared to pre-immune serum; conversely, 18S RNA shows only a 1–2 fold enrichment under the same conditions. These results demonstrate that ITGA6 mRNA is found in VICKZ-containing ribonucleoprotein complexes in chick embryos, suggesting a possible mechanism for how VICKZ may regulate epithelial integrity and the timing of CNC delamination.

### VICKZ Is Not Required in Xenopus CNC Cells for Their Migration

**Fig 7:**
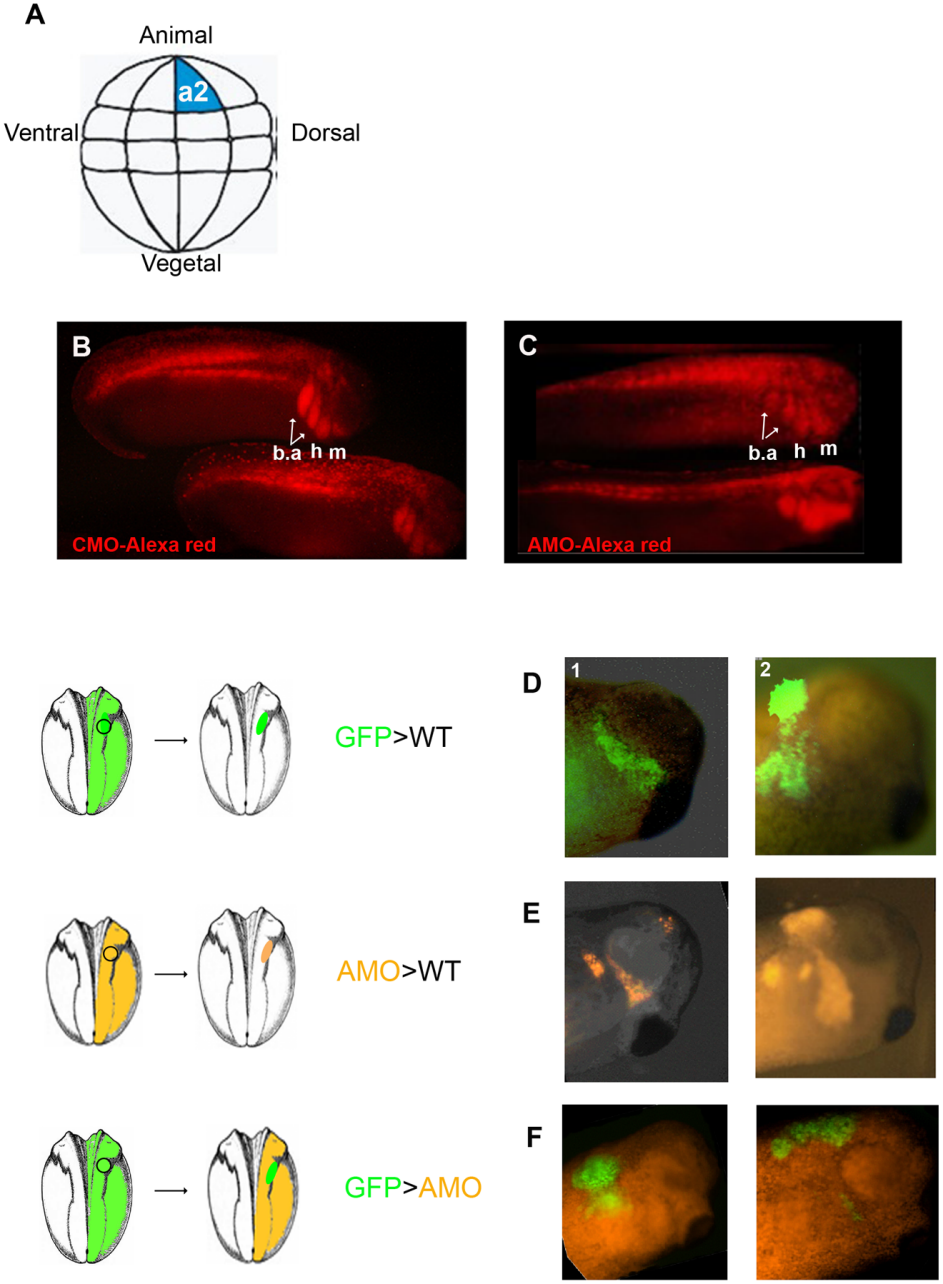
Global reduction of VICKZ in Xenopus embryos inhibits CNC migration non-cell autonomously. (A) A scheme illustrating the position of blastomere a2 in a 32-cell stage embryo that gives rise to most of the CNC cells in the embryo. (B,C) 32 cell-stage embryos were injected in a2 with Alexa-red and either control morpholino (CMO) (B) or xVICKZ antisense morpholino (AMO) (C) and allowed to develop to tailbud stage. Both AMO-and CMO-injected cells reach their homing sites in the branchial, hyoid, and mandibular arches (b.a., h, m). (D-F) CNC orthotopic transplantations. Wild–type CNC grafts (in green) exhibited normal migration in control embryos (D), with grafts taken from a rostral position in the neural folds migrating along the mandibular migration pathway (D1) whereas grafts from more caudal position migrating to the branchial arches (D2). Grafts taken from xVICKZ-depleted embryos in orange, were able to migrate properly in WT embryos (E). However, wild-type CNC (green) were unable to migrate in xVICKZ AMO-injected embryos (orange). Abbreviations: m, mandibular; h, hyoid; b.a, branchial arches.

Our studies in chick show that inhibiting VICKZ expression in prospective CNC cells enhances their delamination. In previous studies on the role of VICKZ in amphibian NC migration, reduction in VICKZ expression by an AMO, throughout the entire embryo, reduced CNC migration [21]. In an effort to understand the source of this difference, we revisited the question of the role of VICKZ in Xenopus embryos using methods that allowed us to reduce VICKZ expression in only a subset of cells. Based on the fate map that has been constructed for 32-cell stage embryos, a2 blastomeres (Fig 7A) give rise to a number of different cell types, including the majority of CNC [32,33]. Accordingly, we found that when 32-cell stage embryos are injected in a2 blastomeres with fluorescinated-dextran, the progeny of this cell populates the branchial arches (a homing site for CNC), as well as the epidermis, brain, and spinal cord (as predicted by the fate map [32,33]; Fig 7B). This distribution of a2 blastomere lineages was unchanged, however, when AMO against xVICKZ (that was previously shown to reduce VICKZ expression by 80%) was injected with the fluorescinated-dextran into the same a2 blastomeres (Fig 7C).

As another approach for studying the effects of reducing xVICKZ expression in only a subset of cells in an otherwise wild type environment, we used a CNC transplantation assay developed to study migratory behavior of CNC cells in *Xenopus* [23]. Donor embryos are injected with either GFP mRNA or xVICKZ-AMO in conjunction with Texas red-labeled dextran into one blastomere of a 2-cell-stage embryo. At stage 14, CNC cells are excised as a block of cells and transplanted orthotopically into host embryos at the same stage. When GFP-labeled CNC cells are transplanted from wild type (WT) embryos into control embryos, approximately 90% of the transplants show migration along the known paths for CNC (Fig 7D, two representative embryos). Similarly, AMO-injected CNC transplanted into control embryos are capable of migration along the same pathways as the controls (Fig 7E, two representative embryos). Transplanted GFP-injected WT CNC grafted onto AMO-injected embryos, however, were mostly unable to migrate in the xVICKZ-reduced embryos (Fig 7F, two representative embryos). Taken together, these results suggest that the reduction in CNC migration originally observed in the AMO-injected Xenopus embryos is a result of a global, non-cell autonomous reduction in VICKZ expression.

## Discussion

### Down-Regulation of VICKZ Is Both Necessary and Sufficient for CNC Delamination

The results presented here indicate that the down-regulation of VICKZ protein in the dorsal neural tube is both necessary and sufficient for cranial neural crest delamination. Although the migratory CNC marker, Snail2, is already expressed in 5ss embryos in the lateral edges of the neural tube at midbrain axial levels (Fig 1F), these cells do not actually undergo EMT and exit the tube until 7-8ss, when VICKZ expression is down-regulated in the tube in precisely these cells (Fig 1G). When the Y396F-GFP VICKZ point mutation, which functions in a dominant negative fashion to inhibit translation of VICKZ targets, is electroporated into 2-4ss embryos, the construct begins to be expressed after about 7 hours (MSC, personal observation). Under the incubation conditions used in these experiments, with a somite formed every 1.5 hours, this corresponds to about the 8ss, concomitant with the onset of CNC migration in the most dorsal region of the neural tube. Thus, expression of the electroporated Y396F construct begins as EMT of the dorsal-most CNC commences, and this expression is sufficient to enhance emigration of the majority of the cells that receive the plasmid. CNC emigration at these axial levels is usually finished by 14-15ss (see Fig 1E), which corresponds to approximately 15 hours post-electroporation. Nevertheless, electroporation of the VICKZ1-GFP construct, which maintains expression of VICKZ protein, prevents EMT of the CNC, even after 20 hours. These results suggest that the down-regulation of VICKZ is also necessary for emigration of CNC.

### A Role for VICKZ in Maintaining Epithelial Integrity-A Proposed Mechanism via Integrin α6

Although 70% of the cells that receive the Y396F construct at 2-4ss exit the tube after 10 hours, 30% remain in the tube. When the electroporated embryos were incubated for 20 hours, however, not only were no GFP-positive cells retained in the tube, but the entire region of the tube that had been electroporated had also dissociated. A similar phenomenon occurred when VICKZ activity was down-regulated by the dominant negative construct in the developing dermomyotome. Here, too, the epithelial structure of the expressing tissue was completely disrupted. It is important to note that the GFP-positive cells in both cases do not look pyknotic or dying, and in fact seem to be migrating either along the normal migration pathways (in the case of the CNC) or into the adjacent mesoderm (in the case of the dermomyotome). These results suggest that the Y396F construct is not toxic to the cells but rather, by inhibiting the activity of VICKZ, is weakening the epithelial integrity of the tissues in which it is expressed.

Because of its inability to undergo phosphorylation by Src kinase, the Y396F construct does not release bound VICKZ targets [26]. In this manner, it functions in a dominant negative fashion with respect to translation [27]. By sequestering bound mRNAs, this construct essentially titrates out these target RNAs. If the VICKZ proteins normally maintain the stability of these RNAs and localize them to the basal side of the tube where they could be translated (following phosphorylation by Src), then overexpressing the Y396F construct would be the equivalent, vis-à-vis target RNAs, of down-regulating endogenous VICKZ levels. One potential target we have identified in the midbrain neural tube is Integrin α6. The expression of Integrin α6 protein mirrors that of VICKZ, not only temporally but also spatially, being expressed in the basal region of the neural epithelium before fusion and down-regulated in the dorsal region of the tube after fusion. Although we do not know where Integrin α6 mRNA is localized within CNC cells, the tight juxtaposition of Integrin α6 protein at the basal membrane with VICKZ protein is consistent with a model of localized RNA giving rise to localized protein (as seen in chick embryo fibroblasts with β actin mRNA and VICKZ1; [34]). Furthermore, Integrin α6 protein expression is mediated by VICKZ: abnormally maintained in the dorsal-most cells in the electroporated hemi-tube that overexpresses VICKZ protein, and down-regulated precociously in the electroporated hemi-tube expressing Y396F. Integrin α6 mRNA is very efficiently immunoprecipitated by pan-VICKZ antibody indicating that it is part of a VICKZ ribonucleoprotein particle. Although Integrin α6 mRNA does not appear to have the bipartite binding site described for ZBP1 target RNAs [35], the full 3’ UTR of the chick messenger mRNA has not yet been identified.

Not only does expression of the dominant negative construct induce EMT in the midbrain neural tube, but prolonged expression of Y396F causes a dissociation of epithelia, both in the neural tube and in the dermomyotome. It is intriguing to note that Integrin α6 expression is induced in many tissues in the developing embryo when cells undergo a mesenchymal-to-epithelial transformation (MET) [36,37]. Indeed, when antibodies recognizing either Integrin α6 or its ligand, the E8 fragment of laminin A, are introduced into organ cultures induced to undergo MET, epithelial structures are inhibited [38,39]. Laminin is present in the basal membrane of the entire developing neural tube but is degraded in the dorsal roof plate region when neural crest cells emigrate [40,41]. In light of our findings here and the observations cited above, we can propose a model in which VICKZ protein mediates Integrin α6 expression that, through its interaction with the basal membrane surrounding the neural tube, could help maintain epithelial integrity before EMT. Down-regulation of VICKZ in the dorsal roof plate cells induces a down-regulation of Integrin α6 in those cells, and these Snail 2-positive, N-cadherin- and ZO-1-negative cells are now able to exit the tube and migrate. Perturbations in VICKZ activity indeed cause corresponding changes in Integrin α6 expression, potentially affecting its mRNA stability or translation (and possibly even its intracellular localization). Certainly Integrin α6 is not the only target of VICKZ in cranial neural crest, and it will be of great interest to perform a general screen for RNAs that are regulated by VICKZ in the dorsal roof plate of the developing neural tube.

### Non Cell-Autonomous Affects of Reducing VICKZ Expression in Xenopus

The original experiments showing a negative role for the down-regulation of VICKZ expression in Xenopus NC migration were performed by reducing VICKZ expression throughout the embryo (injection of both blastomeres at the 2-cell stage). By inhibiting VICKZ activity in a limited number of cells in the developing chick neural epithelium, it has been possible to observe that a reduction in VICKZ expression promotes emigration in prospective CNC cells. We therefore revisited the Xenopus system to determine whether the global knockdown of VICKZ expression in developing frog embryos leads to the inhibition of CNC migration. When WT CNC cells are grafted onto embryos reduced in VICKZ expression, migration was indeed inhibited. CNC cells injected with AMO directed against VICKZ, however, migrated just as uninjected, control CNC cells, in WT host embryos. Likewise, no reduction in the population of the branchial arches was observed when blastomeres that give rise to most of the CNC were injected with VICKZ AMO. These results suggest that there is a non-cell autonomous effect on neural crest migration when VICKZ expression is reduced throughout Xenopus embryos, essentially masking detection of any potential cell autonomous effects. In the chick embryo, where it was possible to either up- or down-regulate VICKZ expression in only a small number of prospective CNC cells prior to EMT, we could observe how VICKZ acts cell-autonomously to prevent emigration by retaining these cells in the neural epithelium.

### VICKZ and Cancer

The down-regulation of VICKZ in CNC cells undergoing EMT is very reminiscent of its expression pattern in rat mammary carcinoma cells (MTLn3) induced to undergo invasion in an *in vivo* invasion assay [19]. In those experiments, VICKZ1 expression was down-regulated 9-fold in the captured invasive cells as opposed to the cells of the primary tumor, and when VICKZ1 was overexpressed in MTLn3 cells, invasion and metastasis were prevented. Consistent with these results are studies examining the affects of manipulating VICKZ1 expression levels in human breast cancer lines (T47D and MDA231); here as well, VICKZ1 expression was inversely correlated with invasion in vitro [18,42]. Given that EMT is considered to be a hallmark of cancer [43], the down-regulation of VICKZ observed in the prospective CNC cells would appear to be reiterated in breast cancer cells undergoing metastasis.

The vast majority of studies, however, that have compared VICKZ expression with survival, metastasis, and disease-free progression in a wide variety of different types of tumors indicate a strong, positive correlation between VICKZ expression and cancer [10,11,13,14,17,44–46]. Even in the case of triple-negative breast cancers, VICKZ3 is considered to be a biomarker associated with a more aggressive phenotype [47,48]. In vitro assay systems as well have suggested a positive role for VICKZ in helping to mediate directed cell movement [49,50]. How does one reconcile these seemingly opposing views of VICKZ function? The role VICKZ proteins play in a given cell is a function of the target RNAs they bind, in conjunction with the constellation of other RNA binding proteins present on these RNAs. Thus, despite being present in two different cell types, the same RNA could be bound by VICKZ in one cell type but not in the other or, even if bound by VICKZ in both cell types, could function differently. Such context-dependent differences in RNA targets and associated proteins are likely to be important considerations in understanding the role a given RNA binding protein plays in a cell.

## Supporting Information

S1 Fig AMO knocks down VICKZ expression in chick mesenchymal cells. AMO was incubated for 48 hours with a chick mesenchymal stem cell line at the concentrations indicated, in the presence of Endoporter (to enhance uptake). Protein extracts were electrophoresed on a 10% SDS-PAGE gel and blotted with anti-panVICKZ and anti-actin antibodies. When normalized to the actin loading control, 2.5 μM AMO reduces VICKZ expression to 44%, and 10 μM AMO to 39%, of control VICKZ expression.
(TIF)

S2 Fig AMO knocks down VICKZ expression in chick embryos. AMO-FITC (AMO) was injected into the midbrain lumen of 2-4ss embryos and electroporated into cells, as described in Materials and Methods. At 5 somite-stage, the embryos were fixed, processed, and sectioned. A midbrain section has been stained for VICKZ expression (cVICKZ) using the pan-VICKZ antibody (A), with the location of the AMO oligo visualized in the green channel (B); the merge is shown in (C). The arrows in (A) indicate cells containing AMO in which VICKZ expression appears to be downregulated.
(TIF)
