# Supplementary figures and images for: A Novel Role for VICKZ Proteins in Maintaining Epithelial Integrity during Embryogenesis
Source: PLoS One. 2015 Aug 28;10(8):e0136408. doi: 10.1371/journal.pone.0136408 (PMC4552865; doi:10.1371/journal.pone.0136408)

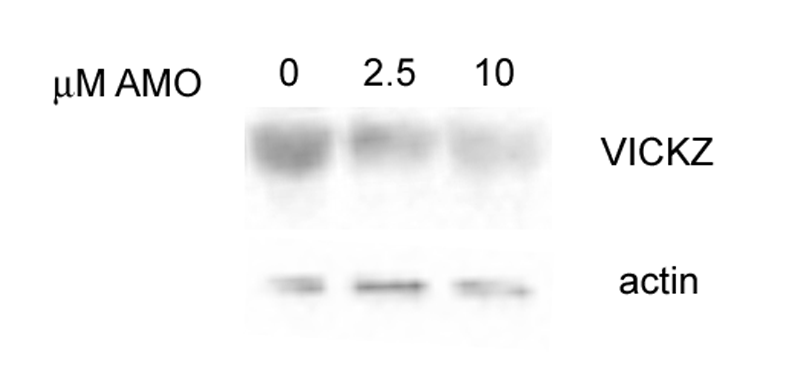

Supplement: S1 Fig — AMO was incubated for 48 hours with a chick mesenchymal stem cell line at the concentrations indicated, in the presence of Endoporter (to enhance uptake). Protein extracts were electrophoresed on a 10% SDS-PAGE gel and blotted with anti-panVICKZ and anti-actin antibodies. When normalized to the actin loading control, 2.5 μM AMO reduces VICKZ expression to 44%, and 10 μM AMO to 39%, of control VICKZ expression. (TIF) [file pone.0136408.s001.tif]

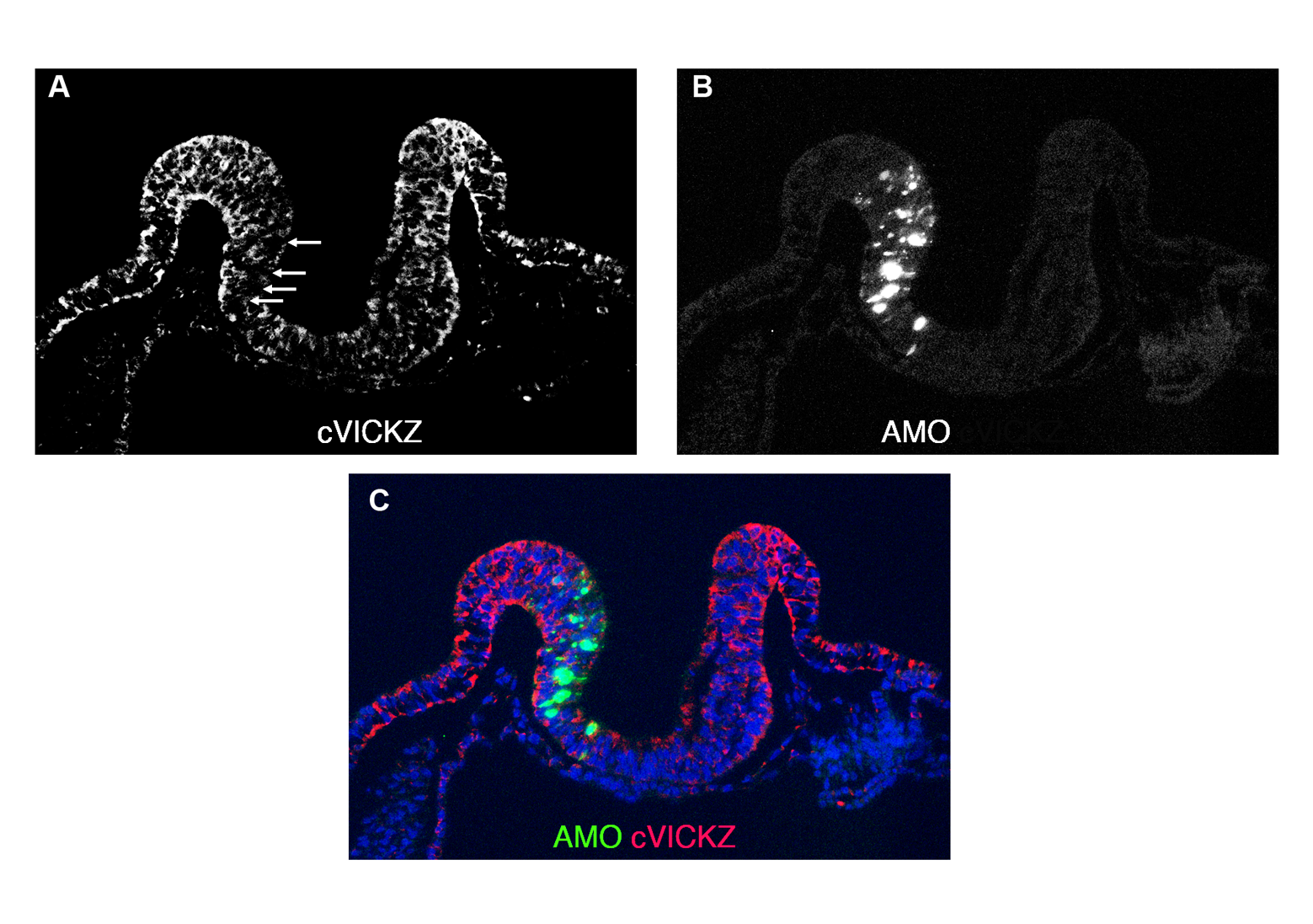

Supplement: S2 Fig — AMO-FITC (AMO) was injected into the midbrain lumen of 2-4ss embryos and electroporated into cells, as described in Materials and Methods. At 5 somite-stage, the embryos were fixed, processed, and sectioned. A midbrain section has been stained for VICKZ expression (cVICKZ) using the pan-VICKZ antibody (A), with the location of the AMO oligo visualized in the green channel (B); the merge is shown in (C). The arrows in (A) indicate cells containing AMO in which VICKZ expression appears to be downregulated. (TIF) [file pone.0136408.s002.tif]
